# 46 Understanding the wants and needs of adults with cerebral palsy who use wheelchairs to participate in community-based physical activity – a qualitative study

**DOI:** 10.1093/eurpub/ckae114.117

**Published:** 2024-09-26

**Authors:** James Czencz, Margaret Wallen, Nora Shields, Christine Imms, Peter H Wilson

**Affiliations:** Healthy Brain and Mind Research Centre, School of Behavioural and Health Sciences, Australian Catholic University, Fitzroy, Australia; School of Allied Health, Faculty of Health Sciences, Australian Catholic University, North Sydney, Australia; Olga Tennison Autism Research Centre, La Trobe University, Bundoora, Australia; Healthy Trajectories: Child and Youth Disability Research Hub, The University of Melbourne, Murdoch Children’s Research Institute, Melbourne, Australia; Healthy Brain and Mind Research Centre, School of Behavioural and Health Sciences, Australian Catholic University, Fitzroy, Australia

## Abstract

**Purpose:**

This study explored the perspectives of adults with CP who use wheelchairs and their support providers regarding physical activity, specifically what they wanted to do and what they needed to participate.

**Method:**

A qualitative study using interpretive description was conducted from a convenience sample of seven adults with CP (5 female, mean age 17.7 years) and five support persons (5 female) who participated in semi-structured interviews. Interviews were conducted using videoconferencing software and were recorded and transcribed. Data were analysed using qualitative data analysis software. Transcripts were checked by the research team. The research team conducted an inductive thematic analysis to identify themes. Consumer partners provided feedback on the project, including interpretation of the themes to ensure relevance to the intended population.

**Results:**

Analysis identified that adults who use wheelchairs strongly desire to engage in physical activity in their community but need inclusive options tailored to their specific needs. Six themes were identified: 1) appreciating diversity among wheelchair users - by recognising individuality, advocating for inclusive activities, and recognising the individual beyond their disability; 2) self-determination and control - emphasising that adults with CP using wheelchairs seek autonomy in their choices and decision-making; 3) flexibility and creativity in adapting activities - participants seek customised, flexible physical activities; 4) accessibility, both physical and social - emphasising the important role of accessible environments, including physical structures and social attitudes, staff training, and knowledge. 5) the crucial role of support providers who hold positions of trust and recognise their recommendations or concerns play an important role in determining the types of activities that adults engage in; and 6) appropriately challenging activities that maximise involvement – ensuring activity and ability are matched carefully, leading to feelings of satisfaction and pride.

**Conclusion:**

Findings highlight the importance of implementing individualised and inclusive approaches to promote physical activity participation among adults with CP who use wheelchairs. To improve participation in physical activity, it is important to not only enhance physical accessibility but also make accommodations that support active involvement by focusing on attitudes, staff training, and listening to the voices of adults with CP.

